# TWEAK Receptor Deficiency Has Opposite Effects on Female and Male Mice Subjected to Neonatal Hypoxia–Ischemia

**DOI:** 10.3389/fneur.2018.00230

**Published:** 2018-04-12

**Authors:** Anton Kichev, Ana A. Baburamani, Regina Vontell, Pierre Gressens, Linda Burkly, Claire Thornton, Henrik Hagberg

**Affiliations:** ^1^Perinatal Brain Injury Group, Centre for the Developing Brain, School of Biomedical Engineering and Imaging Sciences, Kings College London, King’s Health Partners, St. Thomas’ Hospital, London, United Kingdom; ^2^PROTECT, INSERM, Université Paris Diderot, Sorbonne Paris Cité, Paris, France; ^3^Department of Neuroinflammation, Biogen, Cambridge, MA, United States; ^4^Perinatal Center, Institute of Clinical Sciences, The Sahlgrenska Academy, University of Gothenburg, Gothenburg, Sweden; ^5^Perinatal Center, Institute of Neuroscience and Physiology, The Sahlgrenska Academy, University of Gothenburg, Gothenburg, Sweden

**Keywords:** TWEAK, Fn14, perinatal brain injury, hypoxia–ischemia, brain, TweakR, sex differences

## Abstract

Tumor necrosis factor (TNF)-like weak inducer of apoptosis (TWEAK) is a multifunctional cytokine member of the TNF family. TWEAK binds to its only known receptor, Fn14, enabling it to activate downstream signaling processes in response to tissue injury. The aim of this study was to investigate the role of TWEAK signaling in neonatal hypoxia–ischemia (HI). We found that after neonatal HI, both TWEAK and Fn14 expression were increased to a greater extent in male compared with female mice. To assess the role of TWEAK signaling after HI, the size of the injury was measured in neonatal mice genetically deficient in Fn14 and compared with their wild-type and heterozygote littermates. A significant sex difference in the Fn14 knockout (KO) animals was observed. Fn14 gene KO was beneficial in females; conversely, reducing Fn14 expression exacerbated the brain injury in male mice. Our findings indicate that the TWEAK/Fn14 pathway is critical for development of hypoxic–ischemic brain injury in immature animals. However, as the responses are different in males and females, clinical implementation depends on development of sex-specific therapies.

## Introduction

Hypoxia–ischemia (HI) remains an important etiology of brain injury in term infants suffering from intrapartum asphyxia, and in preterms suffering from hypoxic and hypotensive exposures (1, 2). Children from both sexes are at risk of developing brain injury; however, sex differences in the response to HI especially among preterm children are well described (3–5). Most of the available data to date are suggesting that male sex is significant risk factor and male infants are more vulnerable to HI insult.

The pathophysiology is complex and multifactorial. Mechanisms such as mitochondrial dysfunction, oxidative/nitrosative stress, apoptosis, necroptosis, and inflammatory processes are involved (6). Experimental data support the concept that components in the immunoinflammatory reaction contribute to cell death after HI (7). However, our understanding of which inflammatory mediators induce damage of neurons and oligodendroglia precursor cells (OPCs) in neonatal HI is incomplete. It is important to mention that inflammatory responses to injury may be different in males and females as sex differences in microglial response in various neurodevelopment and neurodegenerative disorders have been reported (8–10). This difference is likely to contribute to the differences in outcome after HI insult observed in clinical studies between males and females.

During the inflammatory cascade, infiltrating cells and glial cells resident in the brain (part of both the adaptive and innate immune system) produce reactive oxygen species, release excitotoxic amino acids, pro-inflammatory cytokines, and chemokines (11, 12). Among the prominent cytokine pathways involved in cell death is a subset of the tumor necrosis factor (TNF) family, including TNF-α, TNF-β, FasL, TRAIL, and TWEAK (11, 13–17). We hypothesized that TNF superfamily members participate in the development of injury after HI since several TNF ligand–receptor pairs have been identified in the injurious cascade (18). TNF-α (19) and Fas signaling (13, 20, 21) are already implicated in HI brain injury, but there is limited knowledge about the role of TWEAK in the evolution of neonatal brain injury.

TWEAK is a ubiquitously expressed cytokine and the only confirmed signaling receptor for TWEAK is FGF-inducible molecule 14, Fn14 (also called TWEAKR, TNFRSF12A, and CD266), although another receptor, CD163, has been reported to bind TWEAK (22, 23). Fn14 signaling is mediated by TNF receptor-associated factors (24) and has been inferred to regulate various physiological processes such as cell proliferation, migration, survival, differentiation, and death, depending on the cellular context (25).

TWEAK and Fn14 are expressed at low levels in the brain under normal conditions (15). However, the TWEAK pathway has been implicated in the pathogenesis of several neurological conditions such as ischemic stroke and multiple sclerosis (15, 26–28). Experiments in rodent adult models of stroke showed that inhibition of this pathway by TWEAK neutralizing antibody, soluble decoy Fn14-Fc, or genetic deficiency (26, 27, 29) significantly reduced brain damage after middle cerebral artery occlusion. The beneficial effects of inhibition of TWEAK signaling in this model have been attributed to the increased blood–brain barrier (BBB) stability, which leads to decreased cerebral edema, decreased infiltration of inflammatory cells in the ischemic tissue (30), and decreased neuronal death.

The aim of this study was to test the effect of inhibiting TWEAK signaling in the context of a neonatal model of HI. Here we evaluate changes of the expression of both TWEAK and Fn14 after HI in mice, and the effect of Fn14 genetic deficiency on the development of the brain injury after HI.

## Materials and Methods

### Neonatal HI

This study was approved by the KCL animal ethical committee (AWERB), and all animal experimentation was performed in compliance with the UK Home Office regulations (PPL 70/8376). C57BL/6 wild-type (WT) mice were obtained from Charles River (UK); Fn14 knockout (KO) animals were obtained from Biogen (Cambridge, MA, USA). For the experiments, the homozygote KO females were mated with WT C57BL/6 males to produce heterozygote offspring, those heterozygote animals were mated to produce mixed litters of WT, homo- and heterozygote KO animals which we have used in our experiments.

**Figure 1:**
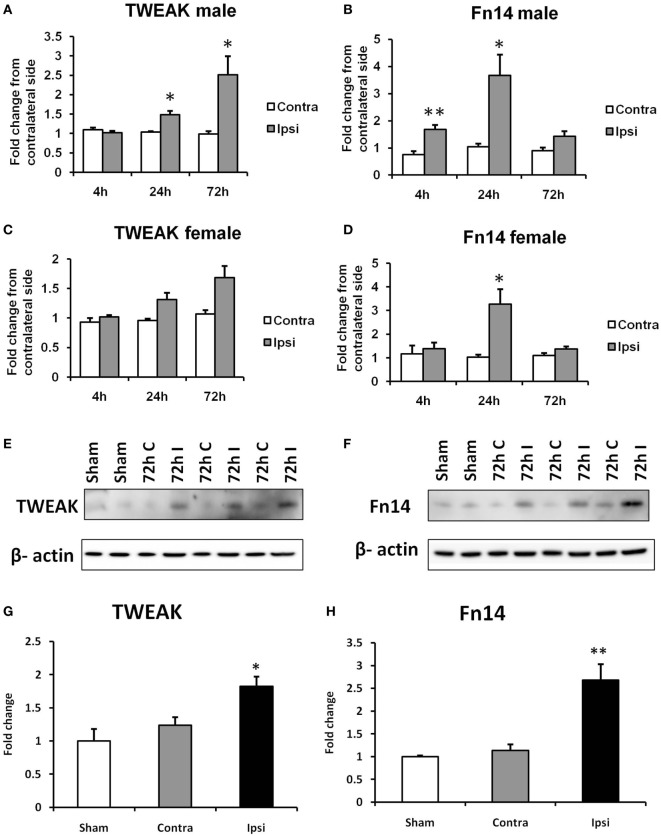
Effects of hypoxia–ischemia (HI) on the expression levels of TWEAK **(A,C)** and Fn14 **(B,D)** in neonatal mice. mRNA levels were measured in contralateral (Contra; white bars) and ipsilateral (Ipsi; gray bars) hemispheres of the brain 4, 24, and 72 h after the insult. The results are expressed as fold change from contralateral side. Western blot detection of TWEAK **(E)** and Fn14 **(F)** protein and densitometry **(G,H)** in sham-operated (Sham, white bars), ipsilateral (I, gray bars), and contralateral hemisphere (C, black bars) brain lysates 72 h after HI. Densitometry of the TWEAK and Fn14 bands was corrected to the corresponding β-actin bands. Bars represent mean + SEM; *p < 0.05; **p < 0.01; versus corresponding contralateral side [*n* = 6–9 **(A–D)** and *n* = 3 **(E,F)**].

**Figure 2:**
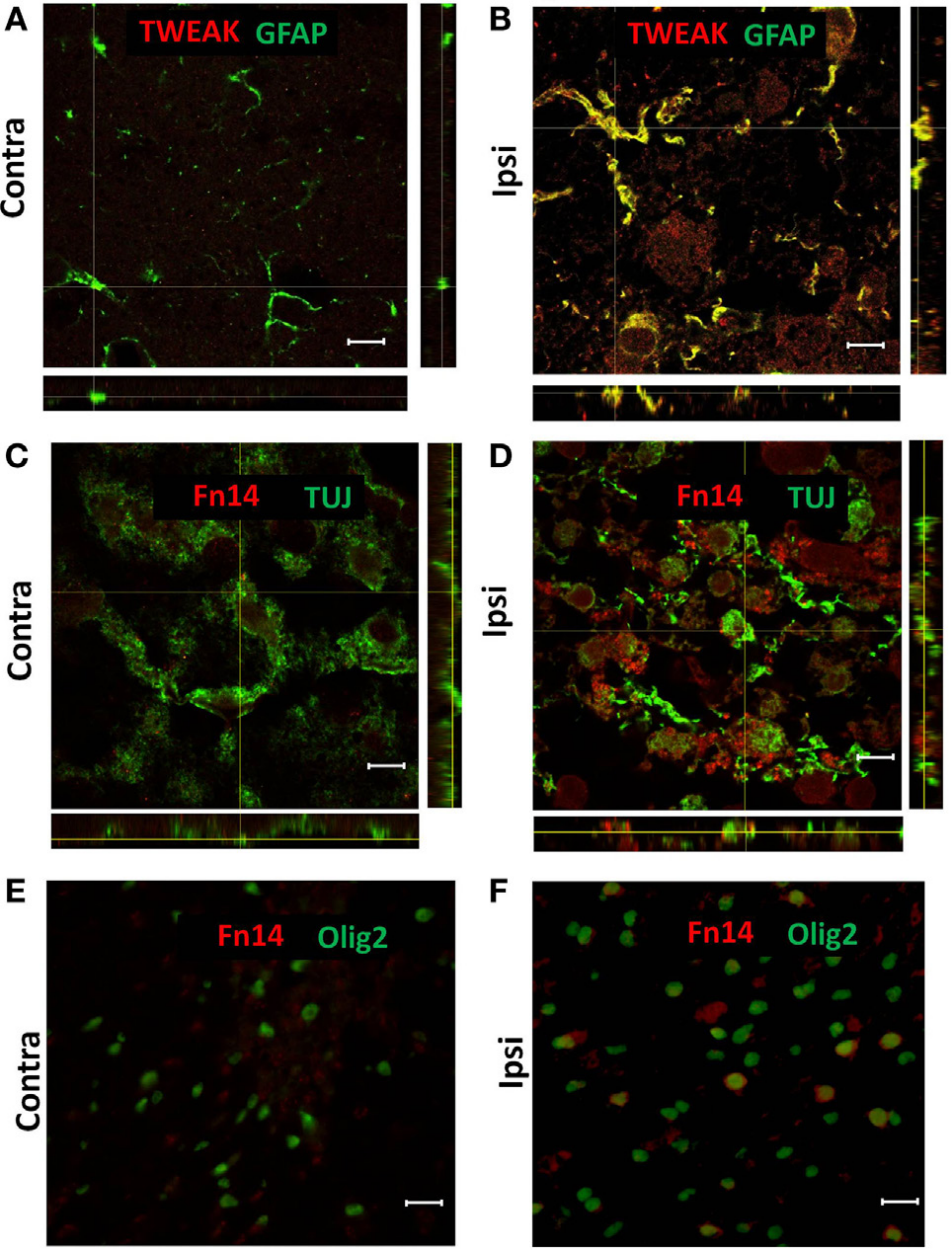
Representative images of confocal **(A–D)** and conventional **(E,F)** fluorescence microscopy of paraffin sections from contralateral [Contra **(A,C,E)**] and ipsilateral [Ipsi **(B,D,F)**] hemispheres of the wild-type mouse brain at 72 h after hypoxia–ischemia. There was TWEAK immunoreactivity (red) in GFAP-positive (green) astroglia **(A,B)** and Fn14 immunoreactivity (red) in TUJ-positive (green) neurons **(C,D)** and Olig2-positive (green) oligodendrocytes (**E,F)**. Scale bar = 10 μm **(A–D)** and 30 μm **(E,F)**.

**Figure 3:**
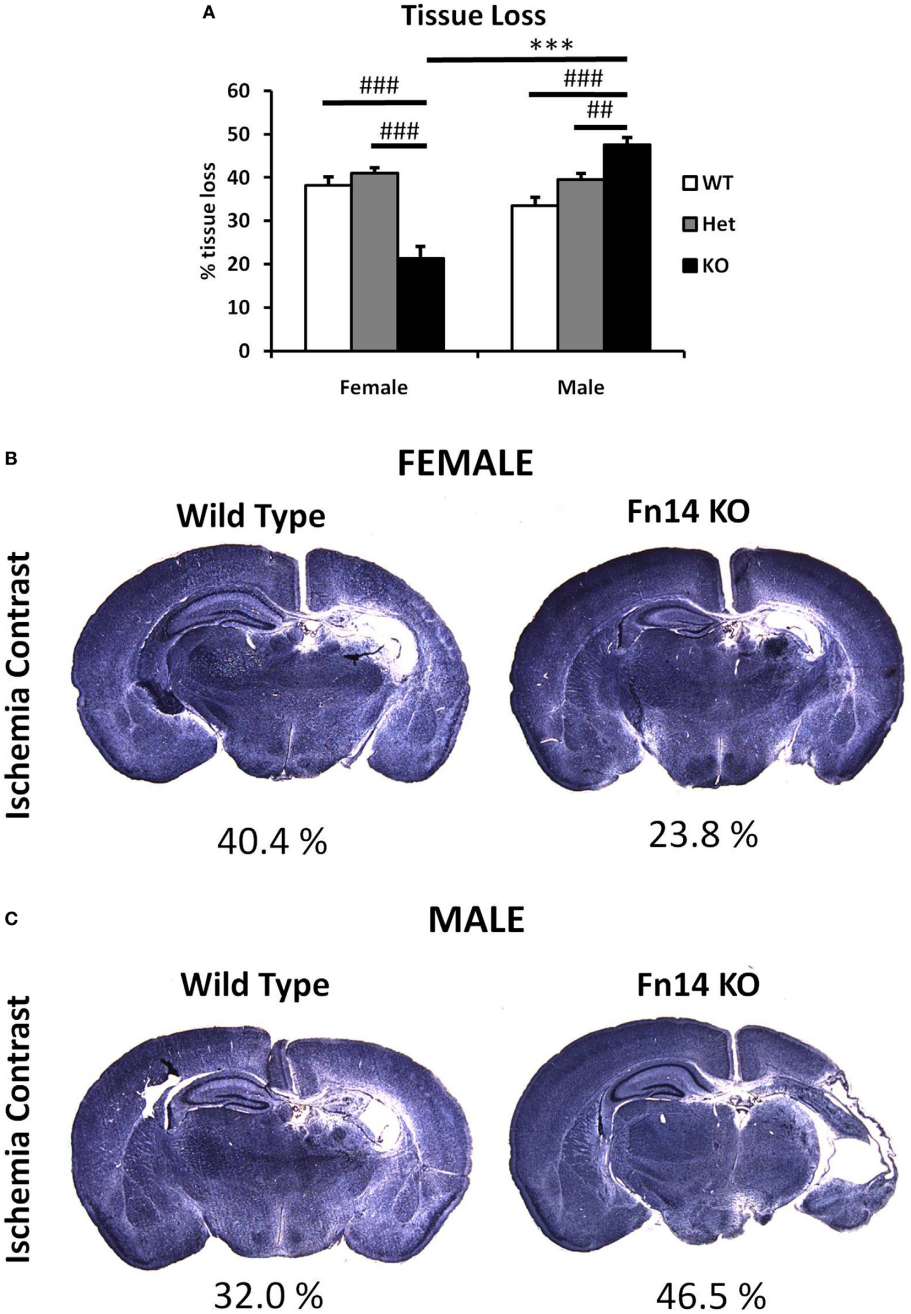
Male Fn14 knockout (KO) mouse pups were more severely injured after hypoxia–ischemia (HI) than females. Brain sections (seven different levels) were generated 7 days after HI and subjected to Ischemia Contrast staining **(A)**. Tissue loss in the ipsilateral side is presented as % loss compared with contralateral across seven levels studied. Bars represent mean + SEM (***p < 0.001, comparison between male and female. ^##^*p* < 0.01 and ^###^*p* < 0.001 for comparison of different genotypes in the same sex group). **(B,C)** Representative images of brain sections from female **(B)** and male mice **(C)** with wild-type (WT) and FN14 KO genotype stained with Ischemia Contrast staining. These images are selected from an animal with tissue loss closest to the average tissue loss for the group. The percent of the tissue loss is indicated below each brain section. WT (white bars), Het (gray bars), and KO (black bars).

**Figure 4:**
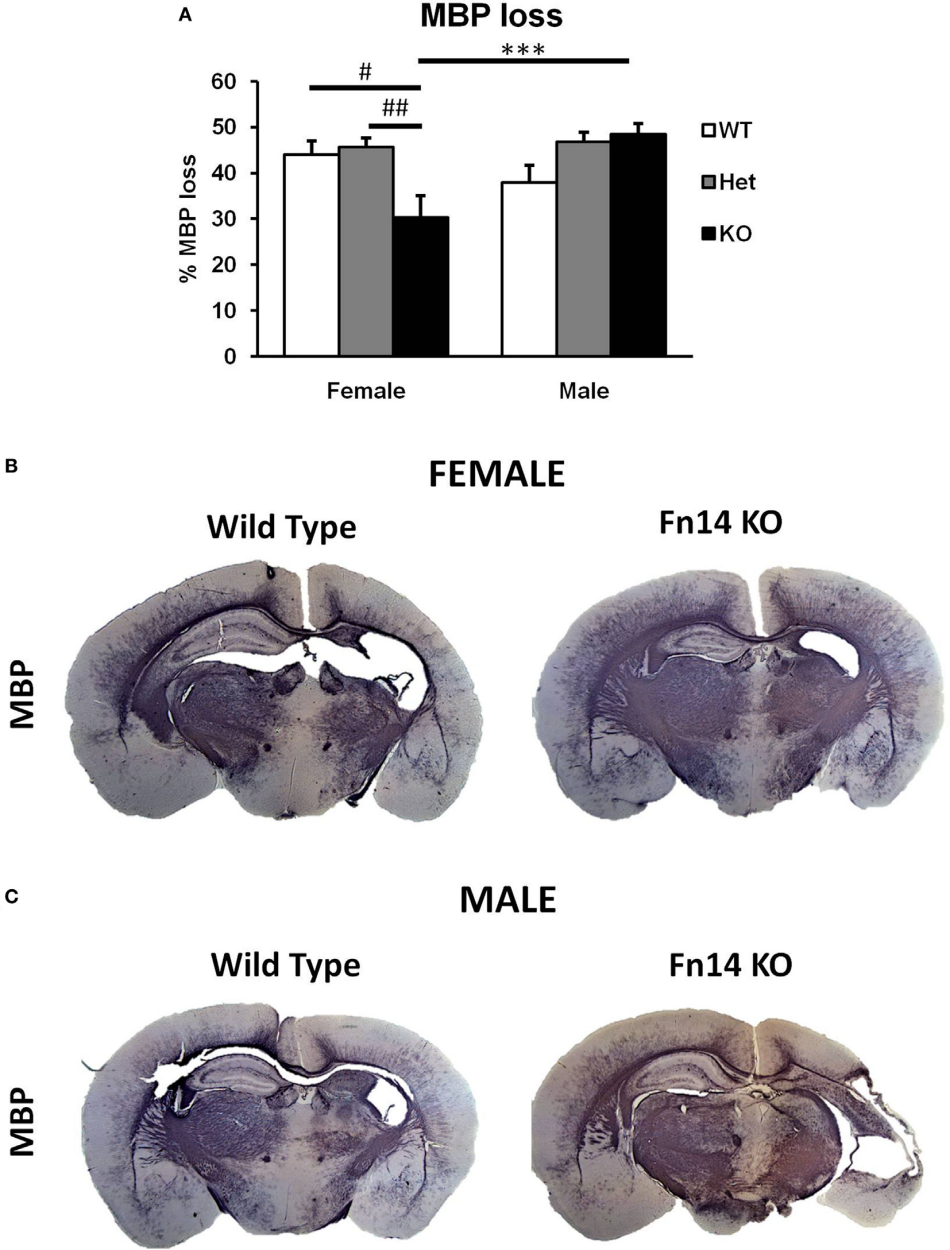
Male Fn14 knockout (KO) mouse pups lost more white matter after hypoxia–ischemia (HI) than females. Brain sections (seven different levels) were generated 7 days after HI and subjected to myelin basic protein (MBP) staining **(A)**. MBP loss in the ipsilateral side is presented as % loss compared with contralateral. Bars represent mean ± SEM (***p < 0.001, comparison between male and female. ^#^*p* < 0.05 and ^##^*p* < 0.01 for comparison of different genotypes in the same sex group). **(B,C)** Representative images of brain sections from female **(B)** and male mice **(C)** with wild-type (WT) and FN14 KO genotype stained with MBP. These images are selected from an animal with MBP staining closest to the average for the group. WT (white bars), Het (gray bars), and KO (black bars).

**Figure 5:**
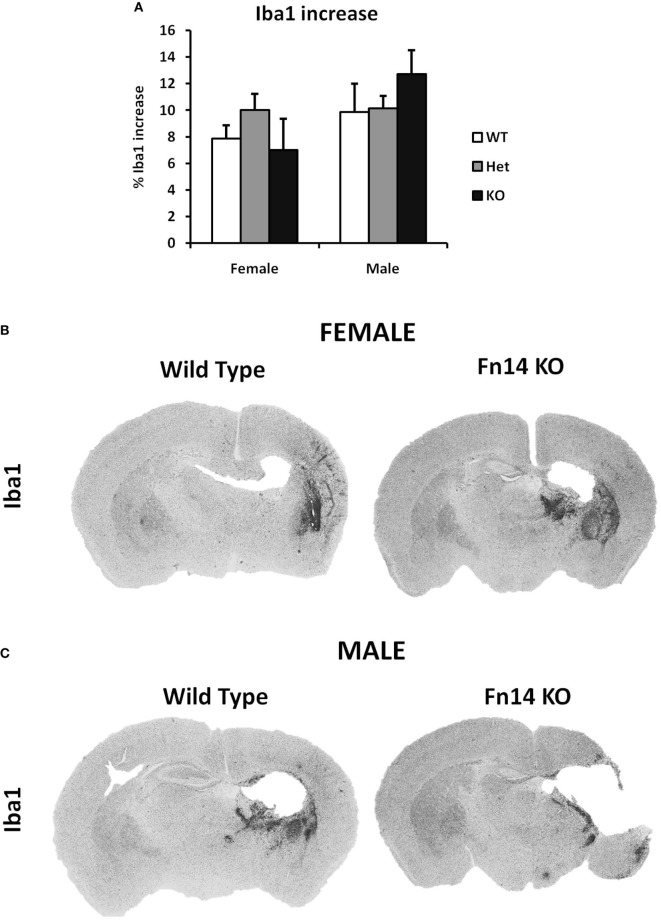
No sex differences were observed in microglia number after hypoxia–ischemia (HI). Brain sections (seven different levels) were generated 7 days after HI and subjected to Iba1 staining **(A)**. Microglial staining in ipsilateral side is presented as percent difference from contralateral side. No significant differences were observed **(B,C)**. Representative images of brain sections from female **(B)** and male mice **(C)** with wild-type (WT) and FN14 knockout (KO) genotype stained with Iba1. These images are selected from an animal with Iba1 staining closest to the average for the group.

**Figure 6:**
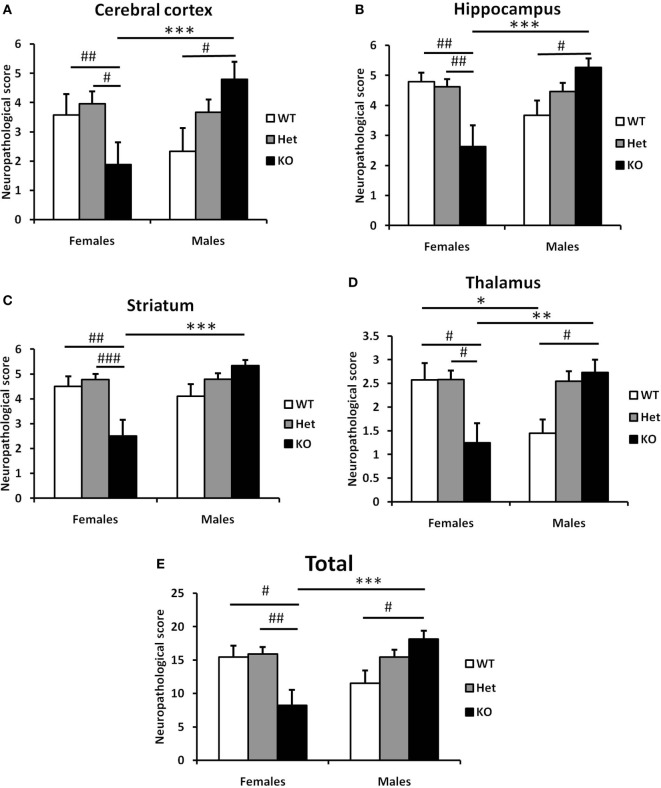
Effect of the genotype on the neuropathological score of the brain after hypoxia–ischemia (HI) in neonatal model of brain injury. Neuropathological scores were assessed from four different brain regions (seven levels generated at 7 days after HI, as above) subjected to Ischemia Contrast and MAP2 staining. The sum of the scores from cerebral cortex **(A)**, hippocampus **(B)**, striatum **(C)**, and thalamus **(D)** were combined for a total score **(E)**. Bars represent mean ± SEM (***p < 0.001, comparison between male and female. ^#^*p* < 0.05; ^##^*p* < 0.01; and ^###^*p* < 0.001 for comparison of different genotypes in the same sex group). Wild type (WT) (white bars), Het (gray bars), and knockout (KO) (black bars).

Neonatal HI was induced at postnatal day 9 according to methods described by Rice et al. (31), but modified for mice (32, 33). Mice of both sexes were anesthetized with isoflurane (3% for induction and 1.5% for maintenance) in nitrous oxide/oxygen (1:1). The left common carotid artery was ligated with Prolene suture (6.0). After the surgical procedure, the wounds were closed and infiltrated with a local anesthetic. After 1 h of recovery with the dam, the pups were placed in a chamber perfused with a humidified gas mixture (10% oxygen in nitrogen) for 65 min at 36°C. The animals were kept in humidified air at 36°C for an extra 10 min before and after the hypoxic exposure. After the hypoxia, the pups were returned to their dams. This procedure results in brain injury in the ipsilateral hemisphere, consisting of cerebral infarction and selective neuronal death in the cortex, striatum, hippocampus, and the thalamus, leaving the contralateral hemisphere undamaged. Sham-operated animals were subjected to isoflurane anesthesia and incision only, without carotid ligation and hypoxia. After the HI procedure pups were killed by decapitation at various time points. Brains were removed and rapidly frozen on dry ice for RNA isolation or fixed in 4% paraformaldehyde for immunostaining. In total, 144 animals were used in this study, 36 for RT-qPCR analysis (Figures 1A–D), 5 for western blot analysis (Figures 1E–H), 3 for fluorescent immunohistochemistry (Figure 2), and 100 for histological evaluation of the brain injury (Figures 3–6).

### Immunohistochemistry

The fixed brains were embedded in blocks of 25 brains, freeze-sectioned (30 μm) and sections collected at 10 different levels using MultiBrain^®^ Technology (NSA Labs, USA). The multibrain sections were stained with MBP, Ischemia Contrast, MAP2 and Iba1 (NSA Labs, USA). For the fluorescent immunohistochemistry, 5 μm paraffin embedded brain sections were prepared, deparaffinized with xylene and ethanol, and pretreated with heating for 20 min in 10 mM citric acid, pH 6.0 with 0.1% Tween 20. Sections were blocked for 20 min with 5% horse serum, 1% BSA in PBS before incubation with primary antibodies overnight at 4°C. We used rabbit anti-TWEAK (Sigma, UK), rabbit anti-Fn14 (Abcam, UK), mouse anti-GFAP (Sigma, UK), mouse anti-Olig2 (Millipore, UK), and mouse anti-TUJ1 (Cambridge Bioscience, UK) antibodies. Immunohistochemistry controls performed with secondary Ab alone (data not shown) or on tissue from Fn14 KO animals with anti-Fn14 antibody (Figure S1 in Supplementary Material) showed no visible staining. For visualization, we used AlexaFluor488-conjugated anti-mouse antibody and AlexaFluor546-conjugated anti-rabbit antibody (Life Technologies). Sections were analyzed using a Leica DM6000 B fluorescent microscope and a Leica TCS SP5 confocal microscope. Images were processed using Leica LAS AF Lite and ImageJ.

### Evaluation of Brain Injury

For our analysis, we have used seven coronal levels across each brain. Total amount of tissue loss was calculated using ImageJ. Briefly, the coronal brain images were segmented by the background and threshold for the Ischemia Contrast staining applied, the hemispheres were manually segmented and intensity of the immunostaining and area calculated for both contralateral and ipsilateral hemisphere. The ipsilateral hemisphere was represented as percentage of uninjured contralateral hemisphere.

Brain injury in different regions was also evaluated by an observer blinded to the study groups, using a semi quantitative neuropathological scoring system (32). Injury in the cerebral cortex was graded both with respect to hypotrophy (0–3) and injury/infarction (0–4; with 0 being no observable injury and 4 being confluent infarction encompassing most of the hemisphere). The damage in the hippocampus, striatum, and thalamus was assessed regarding both hypotrophy (shrinkage) (0–3) and observable cell injury/infarction (0–3), resulting in a neuropathological score for each brain region (0–6) except for cerebral cortex 0–7. The total score (0–25) was the sum score for all four regions.

### Evaluation of Microglia Injury

For our analysis, we have used seven coronal levels across each brain. Total amount of Iba1 staining was calculated using ImageJ. Briefly, the coronal brain images were segmented by the background and threshold for the immunostaining applied, the hemispheres were manually segmented and the immunostaining area calculated for both contralateral and ipsilateral hemisphere. The area of positive Iba1 staining was calculated as percent of hemisphere area. The ipsilateral hemisphere was represented as percentage difference compared with uninjured contralateral hemisphere.

### RNA Isolation

Total RNA from mouse brains was isolated using the RNeasy mini kit (Qiagen) according to the manufacturer’s instructions.

### Quantitative RT-PCR (qRT-PCR)

The reverse transcription reaction was performed using High Capacity cDNA reverse transcription kit (Applied Biosystems) according to the manufacturer’s instructions. qRT-PCR experiments were performed using the StepOnePlus™ Real-Time PCR Systems, TaqMan probes and TaqMan Gene Expression Master Mix (Applied Biosystems). All reactions were conducted in duplicate and corrected to GAPDH expression. Data were analyzed using the delta threshold cycle (CT) method (34).

### Western Blot

30 μg total protein lysate per sample was separated by electrophoresis using 10% Bis–Tris NuPAGE^®^ Novex gels (Invitrogen) and XCell SureLock^®^ Mini-Cell (invitrogen). Proteins were transferred to polyvinylidene fluoride membranes using iBlot^®^ Gel Transfer Device (Invitrogen). Membranes were blocked with 5% non-fat dry milk in Tris-buffered saline containing 0.1% Tween 20 (TBS-T) and immunoblotted overnight at 4°C with anti-TWEAK antibody (sigma) or anti-Fn14 antibody (abcam) diluted 1:2,500 in TBS-T. After washing with TBS-T, membranes were incubated for 1 h with HRP conjugated anti-goat antibody at room temperature. Membranes were washed with TBS-T and developed with SuperSignal West Pico Chemiluminescent Substrate (Thermo Life Science, City, State). The images were taken with ImageQuant™ LAS4000 digital imaging system (GE). Density of the bands was analyzed using FIJI (ImageJ), and the TWEAK and Fn14 bands were corrected to the corresponding β-actin bands.

### Statistical Analysis

The size of samples for our study was calculated using the following formula *n* = (*Z*α/2 + *Z*β)2*2σ 2/δ2, where *Z*α/2 is significance level; *Z*β is power; σ is SD of variable, and δ is minimum important difference. For this study, we have chosen significance level of 5% and power 80%.

The statistical analysis was performed using Prism GraphPad 5.0 software (GraphPad Software). Data are expressed as mean ± SEM. Comparisons between the experimental groups were made using one-way analysis of variance (ANOVA) followed Tukey posttest for comparing between more than two groups with one independent factor (Figures 1G,H) or using two-way ANOVA followed by Bonferroni posttest for comparing between more than two groups with two independent factors (Figures 1A–D, 3, 4, 5 and 6).

## Results

### Neonatal HI Increases Expression of TWEAK and Fn14

The expression of TWEAK and Fn14 was evaluated in a neonatal mouse model of HI. We found a significant increase in TWEAK mRNA levels in the ipsilateral compared with the contralateral hemisphere of male pups at the time points of 24 and 72 h after HI (Figure 1A). In the brains of female animals, we found a similar pattern in TWEAK expression in the ipsilateral side at 72 h after HI that did not reach significance (*p* = 0.052; Figure 1C). Fn14 mRNA expression was significantly induced at 4 h after HI in male animals and 24 h after HI in both male and female animals (Figures 1B,D). Unlike TWEAK where expression increased over time, with highest values at 72 h, Fn14 expression peaked at 24 h and normalized 72 h after HI. At all the time points studied, no differences in TWEAK and Fn14 expression were observed between samples from contralateral compared with sham-operated animals as well as no difference in expression of TWEAK and Fn14 in males versus females in the contralateral hemisphere. Protein expression was analyzed by western blot of brain lysates prepared at 72 h post insult. As observed with the mRNA results, TWEAK and Fn14 protein expression were significantly increased in the ipsilateral hemisphere after HI (Figures 1E–H).

To investigate which cells were responsible for the enhanced expression of TWEAK and Fn14, we analyzed brain sections by immunofluorescence from animals sacrificed 72 h after HI. TWEAK immunofluorescence was strongest in GFAP+ astrocytes in the ipsilateral hemisphere (Figures 2A,B); no co-localization of TWEAK was observed in neurons, microglia, or oligodendrocytes (data not shown). By contrast, Fn14 was expressed in neurons (Figures 2C,D) and in oligodendroglial precursor cells (Figures 2E,F) 72 h after HI. Expression was not observed in any other cell type (data not shown).

### Fn14 Gene Deletion Is Protective in Females but Augments Brain Injury in Males After HI

To examine the impact of TWEAK signaling on the evolution of brain injury, we subjected mice genetically deficient in Fn14 (Fn14 KO) to our neonatal HI protocol and determined the effect of gene KO on brain injury. We found no significant difference in the tissue loss between the female and male WT or heterozygote (Het) animals. However, in female Fn14 KO mice, mean tissue volume loss was significantly reduced compared with WT and Het females [21.3 ± 2.8% in KO females compared with 38.1 ± 2.0 and 41.0 ± 1.4% in WT and Het females, respectively (Figures 3A,B)]. Surprisingly, brain injury was consistently more pronounced in male Fn14 KO compared with WT and Het males [47.5 ± 1.7% in the KO males compared with 33.5 ± 2.0 and 39.6 ± 1.4% in WT and Het males, respectively (Figures 3A,C)]. Thus, there was a differential effect of Fn14 deficiency in female versus male KO mice. To investigate this differential effect further, we analyzed potential changes in white matter [myelin basic protein (MBP)] as well as microglial infiltration (Iba1) after HI injury (Figures 4 and 5). MBP staining was conserved in female Fn14 KO mice (Figures 4A,B), indicating increased white matter integrity, whereas there was a trend toward loss of MBP staining in male Fn14 KO, although this did not reach significance (Figures 4A,C). There were also no significant differences between the genotypes as well as between males and females observed in the sections stained for Iba1 (Figures 5A–C).

Finally, we carried out a detailed analysis of neuropathology scores associated with injury to specific brain regions. In line with our previous observations, we observed differential responses between male and female sections in cerebral cortex (Figure 6A), hippocampus (Figure 6B), striatum (Figure 6C), and thalamus (Figure 6D). In all regions examined, deletion of *Fn14* was associated with attenuation of brain injury in females and increased injury in the male brain (Figure 6).

To discard the possibility of any differences in the brain volume, myelin content, or microglia numbers between males and females and between different genotype groups, we have analyzed and compared contralateral sides of all brains stained with Ischemia Contrast, MBP and Iba1 (Figure S2 in Supplementary Material). We found no significant differences indicating that all differences found in the ipsilateral side were a result of the differences in brain injury.

## Discussion

In this study, we report a sex difference in neonatal HI where Fn14 deletion decreased injury in females and increased injury in males. We have shown that the expression of both TWEAK and its receptor Fn14 is increased in the injured side of the brain after HI *in vivo* and that the increased expression of TWEAK appears to be due to its expression in activated astrocytes. The increase of Fn14 expression was detected mainly in neurons and OPC proximal to the injury site. As neurons and oligodendrocytes are the main cell types lost after HI (35), this may suggest involvement of TWEAK signaling in the development of neonatal brain injury after HI. We also found a marked sex difference in the response to partial or complete *Fn14* gene deficiency. In females, brain tissue loss after HI was reduced in *Fn14* KO mice whereas gene deletion worsened injury in the males (Figures 3A, 4, 5 and 6). Existence of sex differences in the response to HI especially among preterm children is well described (3–5). Sex differences have been shown in HI experiments with neonatal WT mice, where males exhibit more severe brain injury than females (36), although studies using this model do not always find a difference in the extend of brain injury (37, 38). A more common finding is sex-specific responses to various treatments in neonatal animal models, where male and female pups respond differently to treatments administered after injury implicating that different injury mechanisms are at play (37, 39–44). In adult, it is well known that gonadal hormones such as estrogen and progesterone exert protective effect (45, 46). However, in neonatal animals, hormonal influences are less likely to contribute but sex-dependent basic cellular and genetic mechanisms seem to be critical (5).

The interesting finding in our study is that *Fn14* deficiency has an effect on both sexes but is in opposite directions for males and females. The differences that we observed were consistent in all brain areas examined (cerebral cortex, hippocampus, striatum, and thalamus) suggesting that no specific area accounts for the difference but rather a consistent effect across the whole brain.

We currently cannot provide an explanation regarding why TWEAK–Fn14 signaling increases injury in females whereas it is part of a protective response in males. From our experiments, in WT animals, it is obvious that male animals responded differently to HI with an earlier increase in the expression of TWEAK and Fn14 than the females (Figures 1A–D). We and others have previously found differences in the intrinsic apoptotic pathway between males and females (37, 42, 43). It appears that the poly(ADP-ribose) polymerase 1 and apoptosis-inducing factor are more critical components in the caspase-independent apoptotic cascade in neurons of males whereas those of females depend more on the cytochrome C–caspase pathway (37). We can speculate that there are also sex differences in the death receptor pathway that may relate either to the extrinsic apoptotic pathway or RIP1 kinase-dependent necroptosis. The latter has previously been shown to be different in male and female mice after neonatal HI (44). TWEAK treatment induces cell death in primary cortical neurons through NF-κB pathway activation (26, 47). However, NF-κB activation downstream of death receptor activation has been suggested to play a role both in cell survival and cell death depending on context (16, 48), which certainly could be different between the male and female CNS.

Another possible explanation for the sex difference is that TWEAK exerts its neurotoxicity on the neurons and oligodendrocytes not directly through activating their Fn14 receptors but indirectly by changing their environment, for example, through effects on BBB permeability, as observed in adult models of HI injury (30). TWEAK signaling is able to activate matrix metalloproteinase-9 activity in the brain and in primary astrocytes, which in turn increases the permeability of the BBB (49). If the BBB is compromised, this will allow the influx of immune cells from the blood, as well as of inflammatory cytokines and infectious agents, into the brain. In such a case, the state of peripheral inflammatory cells may have a profound effect on the development of brain injury.

We examined the amount of Iba1 positive cells in the brains after HI injury to study whether the sex differences in the injury can be attributed to differences in the microglial immune response. Sex differences in microglial response have been reported in various neurodevelopment and neurodegenerative disorders (8–10). In our study, we have limited our investigation on microglia only to Iba1 staining, which is an estimate for the number of microglia cells rather than their inflammatory status (e.g., secretion of cytokines, phagocytosis, etc.). We did not detect any significant difference in our experiment (Figure 5), meaning that the increase in the number of microglia cell after HI is equal in male and female as well as in different genotypes.

Sex differences in the inflammatory response of astrocytes to inflammation-inducing agents like lipopolysaccharide has been reported (50). This makes astrocytes and their interaction with the neurovascular unit a reasonable candidate for future studies on the sex-dependent role of TWEAK/Fn14 in perinatal brain injury.

More studies are needed to determine whether the sex differences between male and female KO animals can be attributed to differences in the inflammatory response and/or BBB permeability. The other receptor for TWEAK, CD163, is unlikely to contribute for any of the physiological effects observed in our study. CD163 is a scavenger receptor for hemoglobin and haptoglobin without intracellular signaling (51). Moreover, some studies were unable to find any interaction of TWEAK with CD163 (52). A certain degree of promiscuity can be anticipated as it is well known that the members of TNF family have significant structural similarity to each other and it is common that one ligand is signaling through multiple receptors (TNF-α can bind to both TNFR1 and TNFR2; TRAIL can bind to five different receptors in human, etc.). It is not unlikely, however that TWEAK is interacting with and signaling through receptors undescribed to date as a binding partner of TWEAK. Therefore, TWEAK that is present in high concentrations after HI (Figure 1A) could easily bind to other receptors with much lower affinity and trigger some cellular responses. However, to date no alternative binding partners of TWEAK are currently described.

In this study, we have found increase in the expression levels in both TWEAK and Fn14 in the ipsilateral side of the brain following HI and an opposite effect of the Fn14 deletion on the development of the injury for the males and females. These findings are opening different avenues for further investigation of the effects of TWEAK/Fn14 signaling in the context of perinatal brain injury.

## Ethics Statement

This study was approved by the KCL animal ethical committee (AWERB), and all animal experimentation was performed in compliance with the UK Home Office regulations (PPL 70/8376).

## Author Contributions

AK was responsible for design and organization of the study. He performed experiments, data acquisition, statistical analysis, and interpretation and wrote the manuscript. AB performed experiments and data acquisition and reviewed the manuscript. RV performed data acquisition and reviewed the manuscript. LB critically discussed findings and reviewed the manuscript from a technical, statistical, and scientific perspective. PG reviewed the manuscript. CT and HH supported in designing the study, interpretation of the result, critical revision, and preparing the manuscript.

## Conflict of Interest Statement

The authors declare that the research was conducted in the absence of any commercial or financial relationships that could be construed as a potential conflict of interest.

## Supplementary Material

Figure S1 Representative images of confocal **(A,B)** and conventional **(C,D)** fluorescence microscopy of paraffin sections from wild type [WT **(A,C)**] and Fn14 knockout [Fn14 KO **(B,D)**] of the mouse brain. There was Fn14 immunoreactivity (red) in TUJ-positive (green) neurons **(A)** and Olig2-positive (green) oligodendrocytes **(C)** in the WT animals. Lack of any Fn14 staining in Fn14 KO animals **(B,D)**. Scale bar = 10 μm **(A,B)** and 30 μm **(C,D)**.

Figure S2 No difference in total brain tissue **(A)**, myelin basic protein (MBP) staining **(B)**, and Iba1 staining **(C)** between males and females and between wild-type (WT), Fn14 knockout (KO), or heterozygote (Het) animals. Brain sections (seven different levels) were generated 7 days after hypoxia–ischemia and subjected to Ischemia Contrast staining **(A)**, tissue area in the contralateral side was measured and presented as square millimeters. MBP immunostaining **(B)** and Iba1 immunostaining **(C)** of the contralateral side are presented as percentage of total brain area of the contralateral side. Bars represent average + SEM.
