# Supplementary figures and images for: TWEAK Receptor Deficiency Has Opposite Effects on Female and Male Mice Subjected to Neonatal Hypoxia–Ischemia
Source: Front Neurol. 2018 Apr 12;9:230. doi: 10.3389/fneur.2018.00230 (PMC5906546; doi:10.3389/fneur.2018.00230)

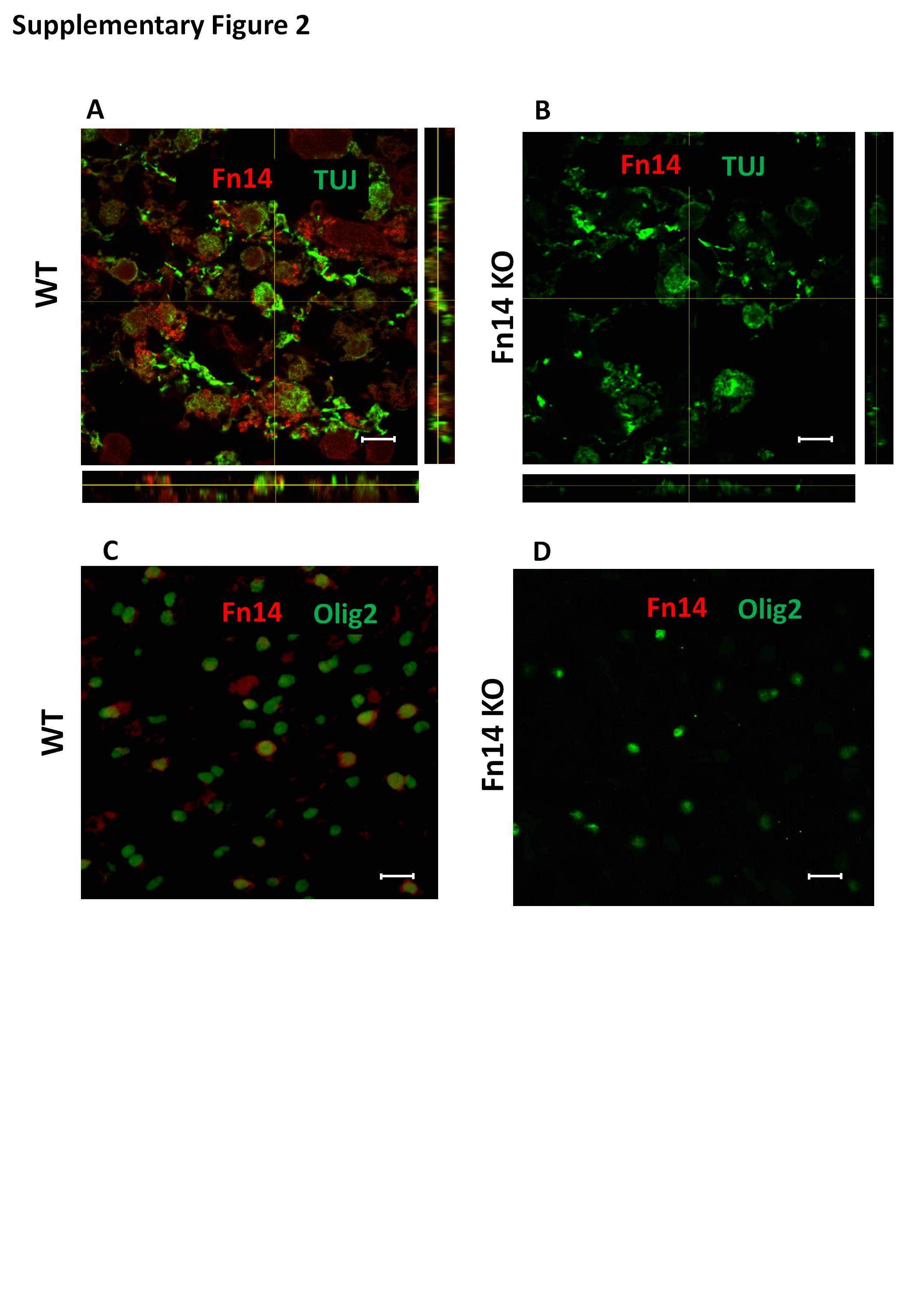

Supplement: Figure S1 — Representative images of confocal (A,B) and conventional (C,D) fluorescence microscopy of paraffin sections from wild type [WT (A,C)] and Fn14 knockout [Fn14 KO (B,D)] of the mouse brain. There was Fn14 immunoreactivity (red) in TUJ-positive (green) neurons (A) and Olig2-positive (green) oligodendrocytes (C) in the WT animals. Lack of any Fn14 staining in Fn14 KO animals (B,D). Scale bar = 10 μm (A,B) and 30 μm (C,D). [file Image_1.jpeg]

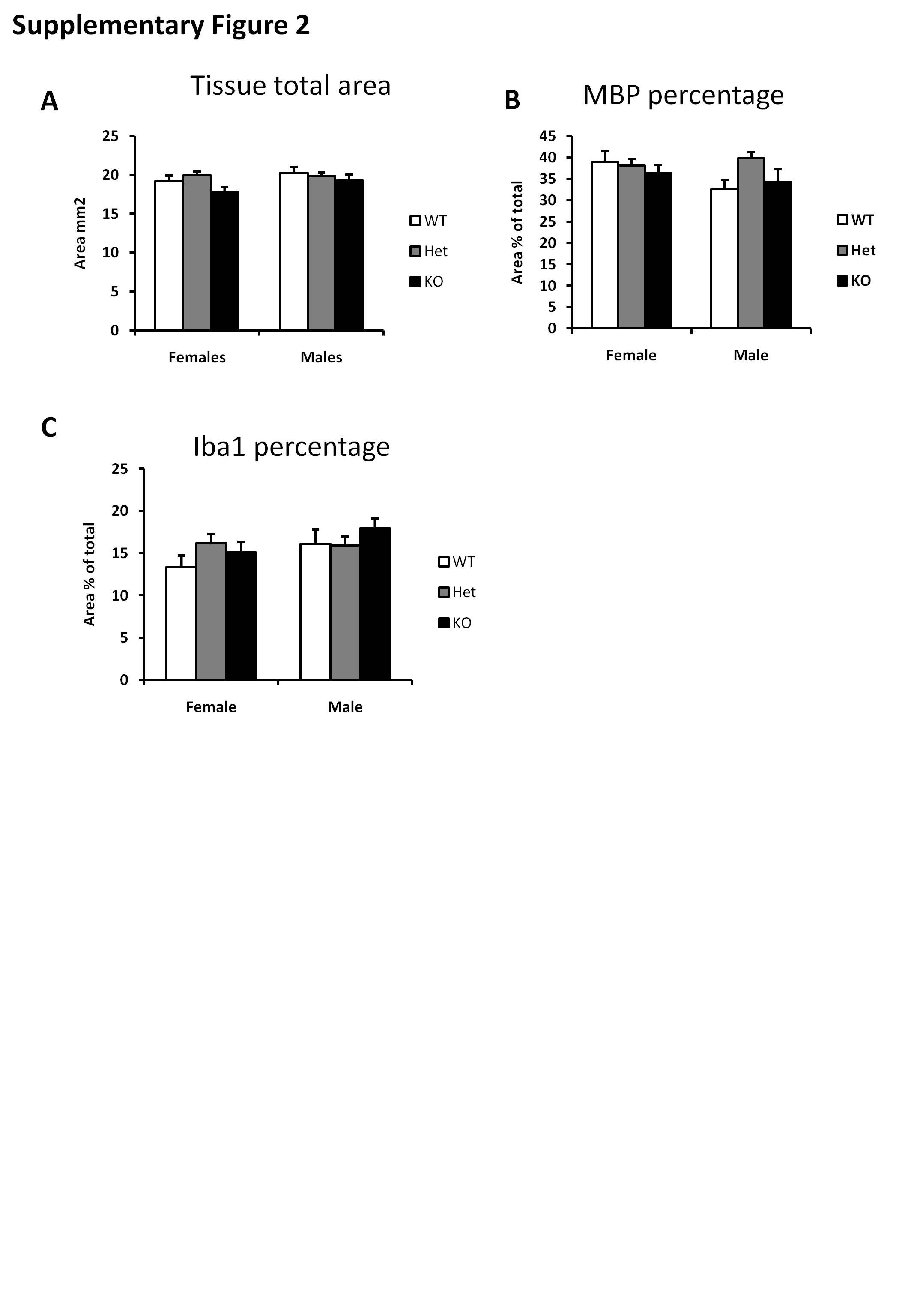

Supplement: Figure S2 — No difference in total brain tissue (A), myelin basic protein (MBP) staining (B), and Iba1 staining (C) between males and females and between wild-type (WT), Fn14 knockout (KO), or heterozygote (Het) animals. Brain sections (seven different levels) were generated 7 days after hypoxia–ischemia and subjected to Ischemia Contrast staining (A), tissue area in the contralateral side was measured and presented as square millimeters. MBP immunostaining (B) and Iba1 immunostaining (C) of the contralateral side are presented as percentage of total brain area of the contralateral side. Bars represent average + SEM. [file Image_2.jpeg]
